# Untargeted GC/TOFMS unravel metabolic profiles in cerebrospinal fluid of Chinese people living with HIV

**DOI:** 10.1002/jcla.23673

**Published:** 2021-01-21

**Authors:** Alim Keram, Ning Pei, Tangkai Qi, Jingna Xun, Yutong Gu, Wenwei Li

**Affiliations:** ^1^ Department of Neurosurgery Shanghai Public Health Clinical Center Fudan University Shanghai China; ^2^ Department of Tuberculosis Shanghai Public Health Clinical Center Fudan University Shanghai China; ^3^ Department of Infection and Immunity Shanghai Public Health Clinical Center Fudan University Shanghai China; ^4^ Department of Scientific Research Center Shanghai Public Health Clinical Center Fudan University Shanghai China; ^5^ Department of Tuberculosis and Orthopaedics Shanghai Public Health Clinical Center Fudan University Shanghai China; ^6^ Department of Neurology Shanghai Public Health Clinical Center Fudan University Shanghai China

**Keywords:** cerebrospinal fluid, HIV‐associated neurocognitive disorder, human immunodeficiency virus, metabonomic, untargeted GC/TOFMS

## Abstract

**Background:**

Metabolic syndrome becomes a focus of clinical cares to people living with HIV (PLHIV) globally. This study aimed to explore the metabolic profiles in cerebrospinal fluid (CSF) of Chinese people living with HIV (PLHIV).

**Methods:**

Cerebrospinal fluid samples from PLHIV and healthy controls were collected from our hospital. Then, the metabolic profiles of CSFs were analyzed PLHIV with healthy individual as the normal controls using the untargeted GC/TOFMS. Following this, kyoto encyclopedia of genes and genomes annotation and pathway analysis were performed to further explore the underlying mechanism of these metabolic alterations in cognitive impairment of PLHIV.

**Results:**

Both PCA analysis and OPLS‐DA had presented that most samples were localized in 95% CI and the gap between control and HIV could significantly separate from each other. Upon this quality control, a total of 82 known metabolites were identified in CSF between PLHIV and healthy controls. Clustering of these metabolites presented that these differentially expressed metabolites could markedly distinguish HIV from healthy controls. Further pathway analyses showed that TCA cycle (citric acid, fumaric acid, lactate, et al.), amino acid (arginine, proline, alanine, aspartate, glutamine, et al.), lipid (cholesterol, butyrate, et al.) metabolisms were significantly changed in CSF of PLHIV, which might affect the cognitive status of PLHIV via affecting neuron energy support, signaling transduction, and neuroinflammation.

**Conclusion:**

Metabolic profiles were significantly altered in CSF and might play key roles in the etiology of cognitive impairment of PHLIV. Further explore the exact mechanism for these metabolic changes might be useful for cognitive impairment management of PHLIV.

## Graphical abstract

CSF samples from 10 HIV‐infected patients and 10 healthy controls were collected. Then, the metabolic profiles of PLHIV CSFs were analyzed with healthy individual as the normal controls using the untargeted GC/TOFMS. Following this, KEGG annotation and pathway analysis were performed to further explore the underlying mechanism of these metabolic alterations in cognitive impairment of PLHIV.

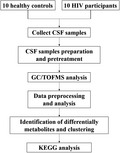

## 1 INTRODUCTION

Human immunodeficiency virus (HIV) is widely recognized as its notorious effect on immune system.^1^ Central nervous system (CNS) is a priority vulnerable orange to HIV infection and long‐term exposure to HIV might develop HIV‐associated dementia (HAD), characterized by motor asthenia and cognitive dysfunction.^2^ Although antiretroviral therapy (ART) could significantly decrease the virus load in blood and cerebrospinal fluid (CSF), the HIV‐associated neurocognitive disorder (HAND) during ART becomes a new challenge and affects 30%‐50% of HIV positive patients presented with during ART.^3^, ^4^ In addition, CNS immune activation can be frequently detected even after a long term of antiretroviral therapy (ART) with suppressed blood and CSF viral load and concerned as contributors for HAND and the exact mechanism remains unknown.^5^, ^6^, ^7^ Moreover, Cassol et al.^8^ have concluded that persistent inflammation, glial responses, glutamate neurotoxicity, and altered brain waste disposal systems in the CSF metabolome of HIV patients on ART contribute to mechanisms involved in HAND. Dickens et al.^9^ consider that worsening cognitive status in HIV‐infected patients is associated with increased aerobic glycolysis in CSF metabolic analysis.

Metabolic syndrome (MetS) is a series of metabolic risk factors, including abdominal obesity, elevated blood pressure, insulin resistance, a proinflammatory or/and prothrombotic state, and become a focus of clinical cares to people living with HIV (PLHIV) globally.^10^ Nowadays, MetS is considered to be a potential risk factor linked to increased HAND in the PLHIV, which is closely associated with the impairments of learning, motion, and execution of patients.^11^ Although Frascati criteria divides HAND into three sub‐disorders: asymptomatic neurocognitive impairment (ANI), mild neurocognitive disorder (MND), and HIV‐associated dementia (HAD), the precise etiology of MetS in HAND remains unclear.^12^

To date, direct sampling of the CNS is largely possible and CSF is usually used to monitored the infectious of CNS. In the current study, CSF samples were collected to explore the variations of MetS in CNS after HIV infection using untargeted GC/TOFMS method followed by bioinformatics analyses. According to these analyses, we hope to provide some new insights and biomarkers in the diagnosis and treatment of neurocognitive disorders of PLHIV.

## 2 MATERIALS AND METHODS

### 2.1 Study design and CSF sample collection

This study was authorized by the Ethics Committee of Shanghai Public Health Clinical Center affiliated to Fudan University. All participants had signed the informed consent. A total of 10 healthy controls (average age ± SD = 42.3 ± 5.3; six males and four females) and 10 HIV participants (average age ± SD = 44.3 ± 4.1; six males and four females) were enrolled in this study and CSF samples of them were collected. Briefly, lumbar punctures were performed with patients according to ADNI protocol^13^ and a total of 12 ml CSF was gathered in polypropylene tubes. Then, tubes were shaken, kept on ice, and immediately stored at −80°C for following analyses.

### 2.2 CSF samples preparation and pretreatment

Before GC/TOFMS, 100 μl CSF samples were removed from −80°C to 1.5 ml EP tubes extracted with 0.5 ml extraction solution (V_ACN_:V_2‐Propanol_:Vwater = 3:3:2) and 5 μl adonitol followed by 30 s vortex mixing and 5 min ultrasonication in ice water. After 10 min centrifugation at 4°C, 3,200 *g*, 500 μl supernatant was transferred to a fresh 1.5 ml EP tube, and 80 μl of each sample was taken out and mixed as quality control (QC) sample. After evaporation in vacuum concentrator, 40 μl methoxyamination hydrochloride (20 mg/ml in pyridine) was added and incubated at 80℃ for 30 min. Following this, 50 μl BSTFA (contained 1%, TMCS, v/v) was added in each sample, mixed, and incubated at 70℃ for 1.5 h. After cooling at room temperature, 5 μl of FAMEs (dissolved in chloroform) was added to QC sample. Then, all samples were subjected to gas chromatograph coupled with a time‐of‐flight mass spectrometer (GC‐TOFMS).

### 2.3 GC/TOFMS analysis

In this study, GC/TOFMS was performed on an Agilent 7890 gas chromatograph system coupled with a Pegasus BT time‐of‐flight mass spectrometer with a DB‐5MS capillary column (30 m × 250 μm × 0.25 μm). As 1 μl aliquot of analyte injected in split mode (10:1), helium was used as the carrier gas with 3 ml/min front inlet septum purge flow and 1 ml/min column flow. Oven temperature ramp was conducted as following condition: 50°C for 0.5 min and raised to 320°C at a rate of 15°C/min and kept for 9 min. Moreover, the front injection temperature, transmission line temperature and ion source temperature were maintained at 280°C, 320°C and 230°C, respectively. The energy of ‐70 eV was non electron collision. The mass spectrometry data were acquired in full‐scan mode with the *m*/*z* range of 75–650 at a rate of 10 spectra per second after a solvent delay of 3.833 min.

### 2.4 Data preprocessing and analysis

MS‐DIAL software was used for raw peaks extracting, baseline correction, deconvolution, peak identification, and peak alignment.^14^ FiehnBinbase database was used for metabolites qualitative, including mass spectrum match and retention index match.^15^ Finally, Peaks were removed in QC sample with ≤50% detection rate or other samples except QC with <50% detection rate or RSD >30% rate.^16^

After processing, SIMCA software (version 15.0.2; Sartorius Stedim Data Analytics AB) was used principal component analysis (PCA)^17^ and orthogonal projections to latent structures‐discriminant analysis discriminant analysis (OPLS‐DA).^18^ Specifically, PCA model was used for the dataset evaluation since it is unsupervised methodology, OPL‐DA, as a supervised methodology, was used to isolate the important metabolites. The first principal component was used to establish OPL‐DA model, and then 7‐folder cross validation was carried out to calculate the values of R2 and Q2. R2 is the ability to explain changes in variables, and Q2 is the ability to predict variables. To check the robustness and predictive ability of the OPL‐DA model, a 200 times permutation tests were further conducted.

### 2.5 Identification of differentially metabolites and clustering

For comparison analyses, traditional univariate analysis (e.g., Student t test and analysis of variance) pay more attention on independent variation of metabolites, while multivariate analysis pays more attention to correlations among metabolites.^19^ Thus, in the current study, Student's *t* test was used to identified the differentially metabolites of CSF between control and HIV samples with *p* < 0.05 and variable importance in the projection (VIP) of OPLS‐DA model >1. Finally, the differentially metabolites were presented in volcano plot and correction among them were estimated using Pearson method and presented with heatmap.^20^

### 2.6 Kyoto encyclopedia of genes and genomes analysis

Metabolism is a comprehensive and dynamic process with multiple pathways involved kyoto encyclopedia of genes and genomes (KEGG) is a commercial database which could integrate gene/genomes, proteins, metabolic pathways to provide an all‐round information for metabolism.^21^ In this study, KEGG database was used to map differentially metabolites to pathway involved in metabolism, providing more information in the metabolism of HIV.^21^ Metabolic regulatory network is a network comprised by metabolic reactions and mechanism involved in these reactions. After acquired metabolites information from KEGG, the regulatory network of differentially metabolites was analyzed and visualized in a network.^22^

## 3 RESULTS

### 3.1 GC/TOFMS analysis

**FIGURE 1:**
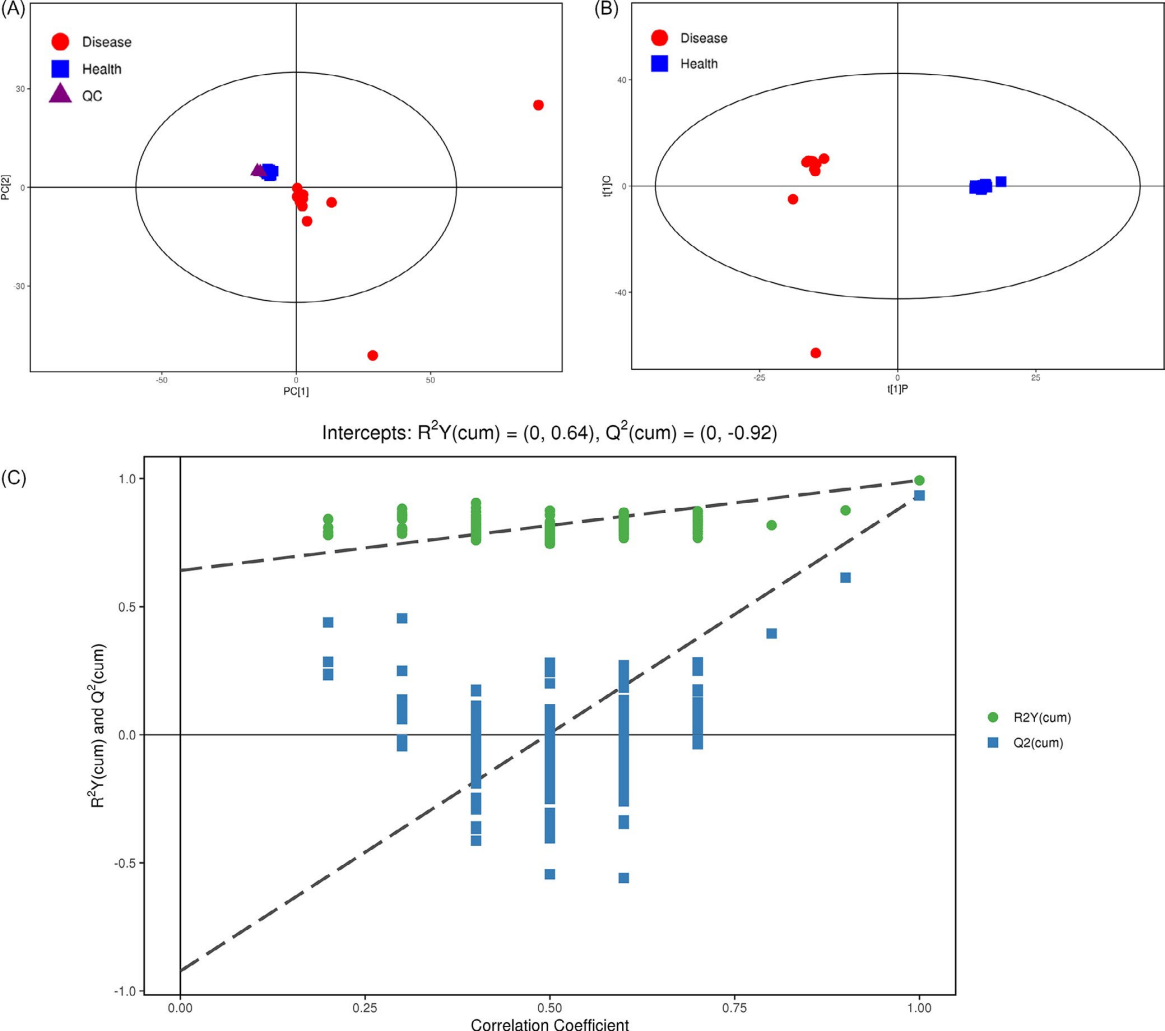
Results for untargeted GC/TOFMS analysis. A, Score scatter plot of PCA model for total samples (including quality control). B, Score scatter plot of OPLS‐DA model for HIV vs. Healthy controls. C, Permutation test of OPLS‐DA model for HIV vs. Healthy controls. CSF, cerebrospinal fluid; OPLS‐DA, orthogonal projections to latent structures‐discriminant analysis discriminant analysis; PCA, principal component analysis; PLHIV, people living with HIV

A total of 1327 Peaks were identified in raw data. After peak filtration and normalization, 1326 peaks were retained. Peak retention time was overlapped well among QC samples and the RSD of internal standard in QC samples ≤ 30% (RSD = 2.94%), indicating that a stability of GC/TOFMS system utilized in this study. PCA analysis showed that most samples were localized in 95% confidence interval (CI; Figure 1A). In addition, OPLS‐DA had presented that most samples were localized in 95% CI and the gap between control and HIV was obvious (Figure 1B). Moreover, the permutation test was also used to validate the model. The R_2_Y of the model was 0.64, which presented a goodness of fit, and the *Q* ^2^ was 0.92, which presented a satisfactory cross‐validation predictive ability (Figure 1C). These analyses showed that majority of testing set samples were correctly classified as healthy control and HIV.

### 3.2 Identification of differentially expressed metabolites between control and HIV

**FIGURE 2:**
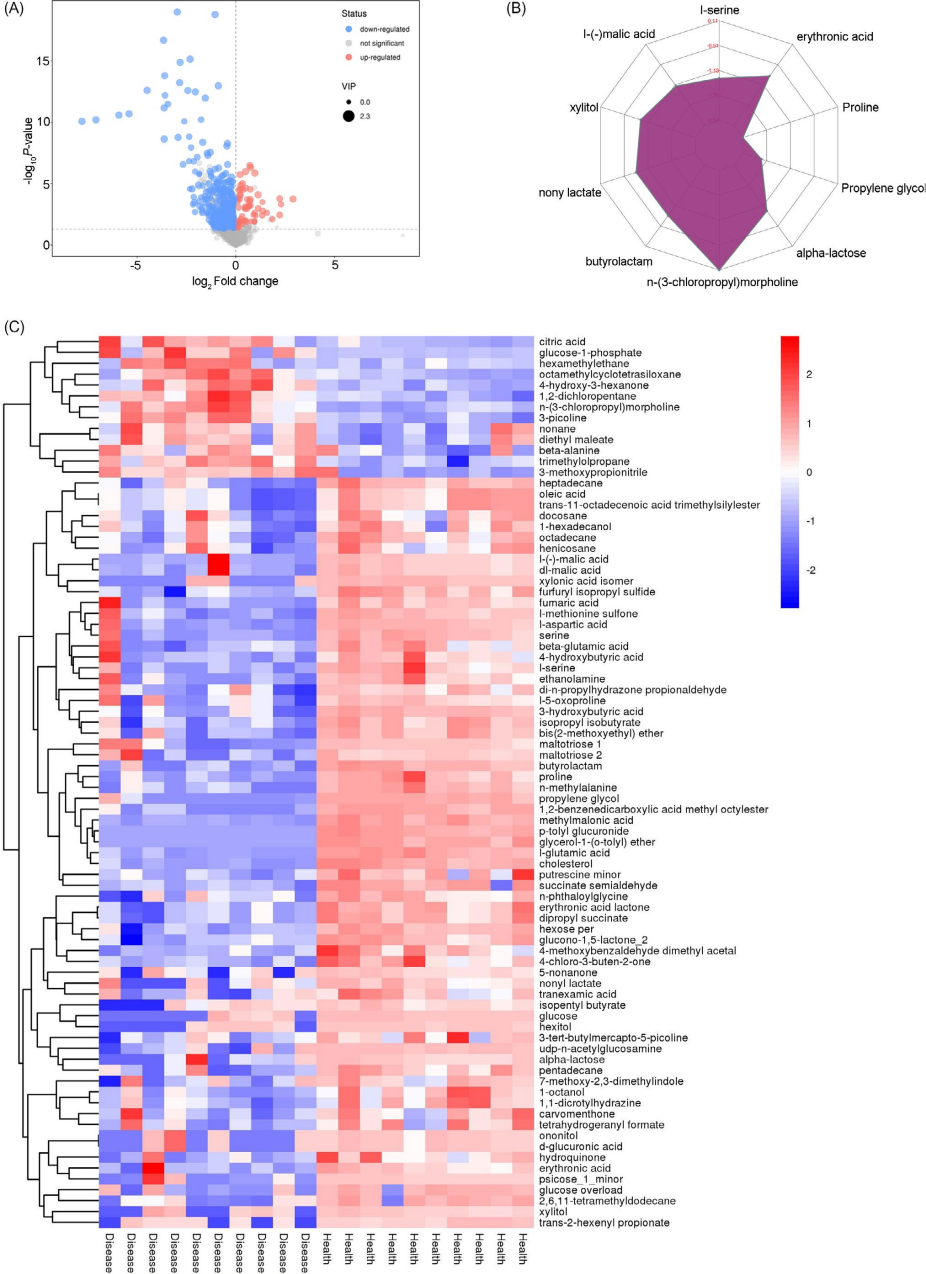
Identification of differentially expressed metabolites in CSF of PLHIV. A, Volcano plot for differentially expressed metabolites between the HIV and Health groups. B, Radar chart analysis for differentially expressed metabolites between the HIV and Health groups. C, Heatmap of hierarchical clustering analysis for differentially expressed metabolites between the HIV and Health groups

After preprocessing, a total of 373 differentially expressed metabolites were identified between the healthy control and HIV. Specifically, 82 of them were known metabolites, including 13 upregulated metabolites and 69 downregulated metabolites. The differentially expressed metabolites were visualized in Volcano plot (Figure 2A). The variation trends of differentially expressed metabolites were presented in a radar chart (Figure 2B) and showed that n‐(3‐chloropropyl) morpholine was most changed metabolites among them. In addition, the correlations among differentially expressed metabolites were also estimated and visualized in heatmap (Figure 2C), suggested that the differentially expressed metabolites could markedly distinguish HIV from healthy control samples.

### 3.3 CSF metabolites and pathways changes

**FIGURE 3:**
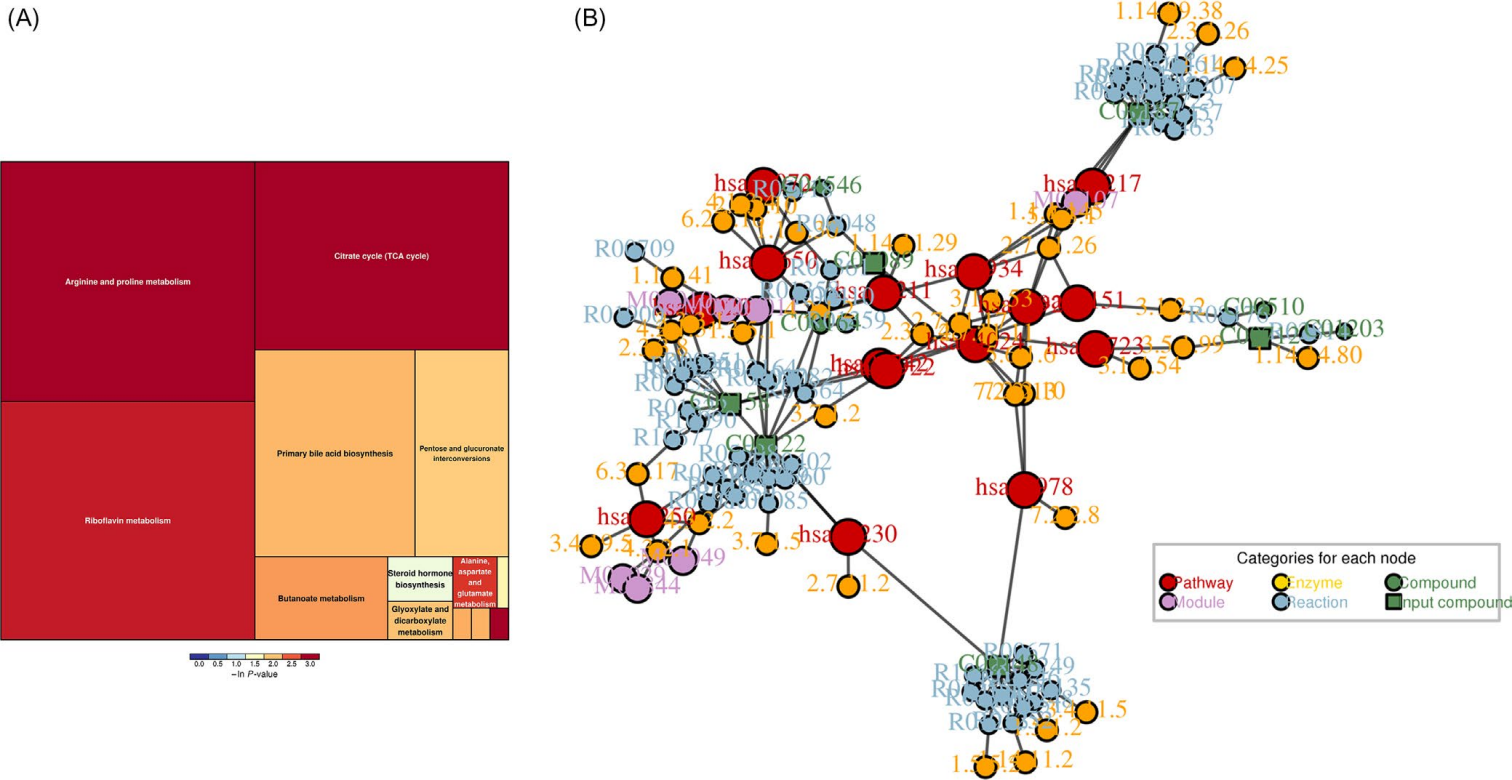
Pathway analysis of differentially expressed metabolites between the HIV and Health groups. A, Pathway topology analysis of differentially expressed metabolites; specifically, each block represent a metabolic pathway, block size positively represents the impact factor of this pathway among this topology analysis, and the color of block positively represents *p* value of this pathway enriched by differentially expressed metabolites. B, Metabolic regulation network analysis of differentially expressed metabolites; specifically, red circle represents a metabolic pathway, yellow circle represents metabolic‐associated enzyme, green circle represents background materials in metabolic pathway, purple circle represents material model information, blue circle represents a chemical reaction, and green square represents the differentially expressed metabolites

For further analysis, the biofunction and mechanism of differentially expressed metabolites were annotated by KEGG and PubChem databases. As a result, a total of 11 KEGG items were significantly enriched by the differentially expressed metabolites, including hsa01100: metabolic Homo sapiens, hsa‐02010: ABC transporters‐Homo sapiens, hsa05230: Central carbon metabolism in cancer—Homo sapiens, hsa00020: Citrate cycle (TCA cycle) ‐ Homo sapiens, hsa00250: Alanine, aspartate, and glutamate metabolism—Homo sapiens (Table 1). Further pathway topology analysis revealed that Citrate cycle (TCA cycle) was the most significant altered metabolic pathway among these metabolites (*p* = 0.0013), followed by Tyrosine metabolism (*p* = 0.019), Arginine and proline metabolism (*p* = 0.019) in turn (Figure 3A). In addition, the metabolic regulatory network was also constructed, including 13 compounds, 44 enzymes, eight modules, 69 reactions, and 15 pathways (Figure 3B ).

**TABLE 1:** KEGG annotation results for differentially expressed metabolites in CSF between HIV and healthy controls

Pathway	Description	Count	Compounds (differentially expressed metabolites)
hsa01100	Metabolic pathways—Homo sapiens (human)	7	D‐Xylitol cpd: C00379; L‐Proline cpd:C00148; (R)‐3‐Hydroxybutyric acid cpd:C01089; Cholesterol cpd:C00187; Citric acid cpd:C00158; Fumaric acid cpd:C00122; Hydroquinone cpd:C00530
hsa02010	ABC transporters—Homo sapiens (human)	3	D‐Xylitol cpd: C00379; L‐Proline cpd:C00148; Amylotriose cpd:C01835
hsa05230	Central carbon metabolism in cancer—Homo sapiens (human)	3	L‐Proline cpd:C00148; Citric acid cpd:C00158; Fumaric acid cpd:C00122
hsa00020	Citrate cycle (TCA cycle)—Homo sapiens (human)	2	Citric acid cpd:C00158; Fumaric acid cpd:C00122
hsa00250	Alanine, aspartate, and glutamate metabolism—Homo sapiens (human)	2	Citric acid cpd:C00158; Fumaric acid cpd:C00122
hsa00350	Tyrosine metabolism—Homo sapiens (human)	2	Fumaric acid cpd:C00122; Hydroquinone cpd:C00530
hsa00650	Butanoate metabolism—Homo sapiens (human)	2	(R)‐3‐Hydroxybutyric acid cpd:C01089; Fumaric acid cpd:C00122
hsa01200	Carbon metabolism—Homo sapiens (human)	2	Citric acid cpd:C00158; Fumaric acid cpd:C00122
hsa01230	Biosynthesis of amino acids—Homo sapiens (human)	2	L‐Proline cpd:C00148; Citric acid cpd:C00158
hsa04922	Glucagon signaling pathway—Homo sapiens (human)	2	Citric acid cpd:C00158; Fumaric acid cpd:C00122
hsa05200	Pathways in cancer—Homo sapiens (human)	2	Cholesterol cpd:C00187; Fumaric acid cpd:C00122

Abbreviations: CSF, cerebrospinal fluid; OPLS‐DA, orthogonal projections to latent structures‐discriminant analysis discriminant analysis; PCA, principal component analysis; PLHIV, people living with HIV.

## 4 DISCUSSION

ART has dramatically ameliorated the life quality and expectancy of PLHIV, but the long‐term exposure to HIV and ART lead to a series of metabolic abnormalities and MetS is frequently detected in PLHIV.^23^ An increasing evidences have suggested that MetS is strongly associated with the increased risk of HAND for PLHIV with suppressed virus load in blood and CSF.^24^ On the other hand, the MetS can also be due to higher MetS risk for that PLHIVs is associated with demographic and behavioral factors, such as sedentary lifestyle and/or absence of leisure activities; having lower education level and/or socioeconomic status; experiencing financial difficulties; or living without a partner.^25^, ^26^, ^27^ Hence, adjustment for socioeconomic factors, rarely included in statistical models, significantly attenuated the MetS–cognition association, highlighting the importance of socioeconomic stratum in identifying and targeting risk factors for an individual's cognitive decline. In the present study, a total of 82 known metabolites were identified in CSF of PLHIV and could significantly distinguish HIV from healthy controls, also suggesting that MetS plays a key role in HIV‐infected CNS among Chinese population.

Brain is one of the most energy sensitive organs in physical and energy support alteration might associated with several neuron‐associated syndromes. According to the untargeted metabolic analyses in this study, TCA cycle is the most significant variated metabolism in CSF of PLHIV and affected other amino acid and lipid metabolism.

Glucose and lactate are both energy source for neuros, but which is the primary energy source remains debated.^28^, ^29^ Lactate is preference synthesized in astrocytes and can shuttle to neurons during increased` synaptic activity via inducing plasticity‐associated genes, including c‐FOS, Zif268, and Arc.^30^, ^31^, ^32^ In addition, Dickens et al.^9^ have documented that accumulation of TCA intermediate metabolite citric acid is markedly associated with worsening cognitive status, and increased lactate is obviously correlated with an improving cognitive status. In the current study, TCA intermediates citric acid was significantly upregulated and fumaric acid was markedly downregulated in CSF of PLHIV than that in the healthy controls, suggested an abnormality activity of TCA in the brain tissue. While, glycolysis product lactate was significantly downregulated in CSF of PLHIV compared with the healthy controls, indicating that a low neuro activity and a worsen cognitive status. Cassol et al.^8^ had also identified that TCA cycle and lactate were significantly dysregulated in CSF of PLHIV, but carbohydrates were attributes to 19% differentially expressed metabolites. We speculated that differences of research subjective population might be the potential cause for MetS difference. Considering of these evidences, we speculated that the imbalance of TCA/glycolysis for neuron energy supply might be responsible for cognitive impairment of PLHIV, and the levels of and their intermediate metabolites or products might be used for further diagnosis. However, the exact mechanism and usage should be further explored.

Amino acids are the cord foundation of proteins and dysregulation of amino acid metabolism is associated with several diseases.^33^, ^34^, ^35^ Chronic inflammation is reported to be closely with the cognitive disorder and amino acid metabolism and immune response are highly cross‐regulated.^36^ Previous study had reported that alterations in amino acid metabolism, such as alanine, aspartate, and glutamine metabolisms, plays critical role in HIV infection‐induced cognitive impairment,^37^, ^38^ which was also consistent with our findings in the current study.

With topology metabolism analysis in this study, arginine and proline metabolism play key role among metabolites in CSF of PLHIV, second by TCA cycle. Arginine is one of major source of nitric oxide which is an important signal regulator in CNS. Due to this preseason, disrupted arginine metabolism has been identified several neurological diseases, such as Alzheimer's disease,^39^ schizophrenia,^40^ and aging.^41^ Neuroinflammation server critical role in cognitive disorder and Galectin‐1 is reported to modulate nitric oxide‐arginase signaling pathway to ameliorate neuroinflammation in HIV‐infected microglia.^42^ Proline is essential to produce connective tissue proteins and proline oxidase (POX) could catalyze proline to pyrroline‐5‐carboxylate and triggers cellular cascades of autophagy and apoptosis via producing reactive oxygen species.^43^ It is reported that HIV‐1 envelope glycoprotein (gp120) could increase the expression and catalytic activity of POX and ROS level in SH‐SY5Y via targeting P53 to promote neuronal autophagy.^44^ In addition, long‐term proline exposure could affect nucleotide catabolism in zebrafish brain.^45^ All these evidences suggested that arginine and proline metabolism might affect the cognitive regulation via modulating the neuro signaling transduction and autophagy/apoptosis.

The brain is highly autonomous in lipid synthesis, and it is generally accepted that neurons could take up astrocyte‐derived lipids to sustain synapse formation and function.^46^ Dyslipidemia is frequently identified in HIV‐infected AIDS patients and lipid metabolism is closely associated with the cognitive status of PLHIV.^47^ In this study, several lipid metabolites were also significantly altered in PLHIV compared with the healthy controls.

In PLHIV, lipid metabolism disorder in CSF might be an important marker for the progression of HAND.^48^, ^49^ Butyrate, a short‐chain fatty acid generated by colon bacterial fermentation, is reported that anti‐inflammatory and memory improvement in several animal models.^50^ Gut microbiome produced butyrate is lowering in the elderly, and butyrate and dietary soluble fiber could ameliorate neuroinflammation in aging mice, suggested that butyrate is associated with aging process.^51^ In this study, butyrate metabolite butanoate is significantly downregulated in CSF of PLHIV. Moreover, pathway analysis showed that 3‐Hydroxybutyric acid and fumaric acid were significantly enriched in butanoate metabolic pathway and topology analysis showed butanoate metabolism serve a critical role in altered metabolism in CSF of PLHIV. Cholesterol is an important source for bile acid synthesis and steroid hormone synthesis. It is reported that high level of high‐density lipoprotein cholesterol was significantly associated with better functional cognitive status in nonagenarians.^52^, ^53^ Moreover, previous meta‐analysis also presented that cholesterol was a risk for dementia and cognitive decline.^54^ In addition, serum total cholesterol and gender were independent predictors of CD4+ cell count, HIV RNA load, and WHO clinical stage.^55^ In this study, cholesterol was significantly downregulated in CSF of PLHIV and primary bile acid biosynthesis acts an important topology status in the metabolism of CSF in PLHIV. Moreover, n‐(3‐chloropropyl) morpholine was the most changed metabolites, we supposed that it should be a metabolin of some specific drug which cannot be determined. According to these findings, we speculated that lipid metabolism, especially cholesterol and butyrate associated metabolisms, could modulate the neuroinflammation to affect cognitive status in PLHIV.

There were still some limitations included in this study. First, although multiple differentially metabolites as well as their associated with pathways and enzymes, the exact mechanism of metabolites regulation remains unclear due to limited research fund. Second, due to incomplete cognitive data, the associations between cognitive status and different metabolites were not assessed in this study. Despite these, findings in this study also provided us some new perspective on MetS information in PLHIV of Chinese population.

In summary, we have explored the metabolism alteration in CSF of PLHIV in China with untargeted GC/TOFMS method. In this study, 82 known metabolites were identified in CSF of PLHIV and could significantly distinguish HIV from healthy controls. Further metabolic analyses showed that metabolic alterations might impact the cognitive status via several paths. Specially, imbalance of TCA/glycolysis for neuron energy supply might directly affect the neuro activity in CNS; abnormal amino acids, such as arginine and proline metabolisms, might influence cognitive activity via modulating the neuro signaling transduction and its autophagy/apoptosis; while lipid metabolism, especially cholesterol and butyrate‐associated metabolisms could affect cognitive status in PLHIV via responding to the neuroinflammation induced by HIV. These findings might provide a directly understanding of the metabolism alterations in CSF, and offer some new insights in the management of MetS‐associated cognitive impairment of PLHIV.

## CONFLICT OF INTEREST

The authors declare that they have no competing interest.

## Funding information

This study was supported by the Hospital‐level project of Shanghai Public Health Clinical Center affiliated to Fudan University.

## Data Availability

The datasets used or analyzed during the current study are available from the corresponding author on reasonable request.
